# From brain to eye: Annexin A1 and A2 as a key mediator of retinal neuroinflammation in Parkinson’s and Alzheimer’s disease

**DOI:** 10.1186/s40942-025-00786-y

**Published:** 2026-02-04

**Authors:** Luiz Philipe de Souza Ferreira, Caio Vinicius Saito Regatieri

**Affiliations:** 1 Department of Morphology and Genetics, Escola Paulista de Medicina, Universidade Federal de São Paulo, Rua Botucatu, 740, São Paulo, SP 04023-900 Brazil; 2 Department of Ophthalmology and Visual Sciences, Escola Paulista de Medicina, Universidade Federal de São Paulo, Rua Botucatu 821, Vila Clementino, São Paulo, SP 04023- 062 Brazil

**Keywords:** Microglial activation, Astrocytes, Annexins, Neuroinflammation

## Abstract

The retina, as an extension of the central nervous system, is highly susceptible to inflammatory and degenerative changes similar to those brain seen in Parkinson’s disease (PD) and Alzheimer’s disease (AD). Annexin A proteins, particularly annexin A1 (AnxA1) and annexin A2 (AnxA2), may play key roles in regulating neuroinflammation and maintaining retinal homeostasis. AnxA1 provides strong anti-inflammatory and pro-resolution actions, limiting cytokine release and promoting a reparative microglial phenotype. AnxA2, which often acts as an S100A10-AnxA2 complex, supports inflammatory resolution through tPA- and TLR4-dependent pathways and contributes to glial stability. In PD, retinal degeneration mirrors brain pathology, and reduced AnxA1 activity may impair the resolution of inflammation. In AD, decreased AnxA1 signalling compromises β-amyloid clearance and maintains chronic neuroinflammation. However, endogenous release of AnxA1 or therapy with AnxA1-derived peptides is capable of promoting efficient phagocytosis by microglia, clearing the neurotoxic environment in AD brains. Overall, it is possible that coordinated AnxA1/AnxA2 activity may mitigate retinal damage, improve the removal of toxic aggregates, and preserve vision, highlighting annexin pathways as promising therapeutic targets for retinal degeneration in PD and AD. This makes it an interesting target for study during retinal degeneration in PD and AD.

## Introduction

The retina, as an extension of the central nervous system (CNS), is particularly vulnerable to inflammatory and degenerative processes, which may originate in the retina itself or reflect neurodegenerative changes in the brain in diseases such as Parkinson’s (PD) and Alzheimer’s disease (AD) [1]. Chronic brain inflammation can induce retinal stress, triggering neuronal death processes, compromising retinal integrity, and contributing to progressive visual deficits [2, 3]. Recent evidence highlights the fundamental role of annexin A (AnxA) proteins Ca²⁺-dependent phospholipid-binding molecules in the regulation of neuroinflammation. In particular, annexin A1 (AnxA1, 37 kDa) and annexin A2 (AnxA2, 36 kDa) have emerged as key modulators of neuroinflammatory signalling in PD and AD. Their actions are mainly mediated by pro-resolution microglial pathways that drive the transition from a pro-inflammatory state to an anti-inflammatory and neuroprotective state [4–7].

## Main body

A key protein in this context is AnxA1, which has received particular attention for its central role in orchestrating the resolution of inflammation. Acting through its interaction with formyl peptide receptors (FPRs), AnxA1 exerts potent anti-inflammatory and pro-resolving effects by suppressing pro-inflammatory cascades. Within the immune system, AnxA1 plays a crucial role in modulating leukocyte responses, promoting cell recruitment to enhance polarization and phagocytic efficiency, as observed in peripheral macrophages [8]. These functions extend to the CNS, where endogenous AnxA1 is recruited in response to brain tissue injury. In microglia, the nuclear translocation of AnxA1 drives pro-resolving responses by suppressing the transcription of inflammatory genes, thereby attenuating the production of pro-inflammatory cytokines such as IL-1β, TNF, and IL-6, and promoting the polarization of microglia from a pro-inflammatory microglia phenotype toward an anti-inflammatory microglia state, which supports efficient phagocytosis [4].

While AnxA1 plays a pivotal role in orchestrating the resolution of neuroinflammation, AnxA2 contributes to cellular homeostasis through distinct but complementary molecular mechanisms. AnxA2 primarily interacts with S100A10, a member of the S100 family of Ca²⁺-binding proteins, forming a dimeric complex composed of two 11 kDa subunits. S100A10 is typically found associated with its AnxA2 ligand as a heterotetrameric S100A10-AnxA2 complex on the cell surface. This interaction enables AnxA2 to exert pro-resolving effects during both acute and chronic inflammatory processes, enhancing microglial activity through S100A10 signaling. Moreover, the interaction of the S100A10-AnxA2 complex with tissue plasminogen activator (tPA) facilitates reactive microglial activation, serving as an important modulator of both pro- and anti-inflammatory responses [6]. Evidence suggests that these pathways extend to the retina, particularly under neurodegenerative conditions [9]. Beyond microglia, AnxA1 also plays a key regulatory role in astrocytes, where its activation has been shown to reduce the release of pro-inflammatory cytokines, including IL-1β, TNF-α and MCP-1, an effect associated with suppression of p38 and JNK-MAPK pathways, but independent of ERK–NF-κB signaling. In parallel, AnxA2–S100A10 signaling modulates astrocytic physiology, influencing neuronal excitability and shaping astrocyte activation states [4, 10, 11]. Together, AnxA1 and AnxA2 orchestrate complementary, cell-type-specific mechanisms in astrocytes and microglia, reinforcing neuroimmune balance within the CNS. Importantly, these pro-resolving actions also occur in the retina [4, 12–14], suggesting that annexin dysregulation in glial cells may contribute to retinal inflammatory degeneration relevant to PD and AD.

In PD, retinal neurodegeneration mirrors brain pathology, including dopaminergic cell loss, α-synuclein (α-syn) aggregation, and chronic microglial activation [2, 15]. In line with findings from substantia nigra microglia, reduced AnxA1 activity in retinal microglia could lead to sustained pro-inflammatory signaling, increased oxidative stress, and enhanced neuronal vulnerability. Conversely, exogenous activation of AnxA1 using the mimetic peptide Ac2–26 may promote microglial polarization toward a pro-resolving phenotype, decrease the expression of pro-inflammatory mediators, and protect retinal dopaminergic neurons from degeneration, paralleling neuroprotective effects observed in cerebral ischemic models [16, 17].

Building upon this retinal perspective, evidence from brain pathology PD models further reinforces the relevance of AnxA1 dysregulation to disease mechanisms. AnxA1 is consistently implicated in these processes: increased microglial AnxA1 immunoreactivity has been observed in post-mortem PD brain’s, and cellular models demonstrate that AnxA1 dysregulation contributes to dopaminergic vulnerability, while its overexpression reduces ROS, inflammatory mediators, and NF-κB activation. Although pathogenic *ANXA1* mutations are rare in PD, functional alterations appear to arise from inflammatory dysregulation rather than genetics [4]. These considerations support the hypothesis that restoring AnxA1 signaling in the PD retina may provide anti-inflammatory and neuroprotective benefits, potentially mitigating visual deficits and preserving retinal integrity, and position AnxA1 as a promising therapeutic target for retinal homeostasis in during the course of PD.

Moreover, AnxA2 also supports anti-inflammatory polarization and phagocytic clearance, indicating that coordinated AnxA1-AnxA2 regulation may help preserve retinal integrity in PD. Studies indicate that the S100A10-AnxA2 complex, or AnxA2 alone, promotes the activation of macrophages and microglia into a pro-resolution phenotype, stimulating anti-inflammatory functions through S100A10-tPA-plasmin and TLR4-modulating pathways [18, 19]. This mechanism is particularly relevant in retinal degeneration associated with PD and AD, both characterized by chronic inflammation, elevated IL-1β, and the accumulation of α-syn, Aβ and Tau. Through its ability to enhance phagocytic activity and facilitate the clearance of these pathological aggregates, AnxA2 may contribute to the resolution of the inflammatory retinal microenvironment [2, 3]. Importantly, AnxA1 and AnxA2 appear to act in a complementary manner, engaging distinct but interconnected pathways-FPR2-dependent pro-resolving signaling for AnxA1, and S100A10/tPA-dependent modulation of phagocytosis for AnxA2-ultimately converging on efficient microglial clearance, inflammation resolution, and neuroprotection.

In AD, reduced or dysregulated AnxA1 signaling leads to persistent microglial activation, impaired Aβ clearance, and increased neuronal vulnerability. Experimental studies demonstrate that AnxA1 deficiency amplifies neuroinflammation and accelerates amyloid pathology, whereas its pharmacological activation with the mimetic peptide Ac2–26 promotes inflammation resolution, enhances microglial Aβ phagocytosis, and protects neurons from degeneration. AnxA1 facilitates Aβ clearance through two complementary pathways: upregulating neuronal neprilysin to reduce amyloid burden and enhancing microglial Aβ uptake for efficient removal [20, 21]. These mechanisms likely operate in the retina, where Aβ accumulation is well documented in AD models and patients [22–25], positioning AnxA1 as a potential retinal protector.

### Future perspectives

Building upon this evidence, it is plausible that coordinated regulation of AnxA1 and AnxA2 signaling in the retina could mitigate inflammatory damage, promote the clearance of toxic protein aggregates, and preserve both visual and retinal neuronal function in PD and AD. Consequently, therapeutic strategies targeting these AnxAs may represent a promising avenue for controlling retinal neuroinflammation and fostering early interventions in neurodegenerative diseases.

In this context, the dual biological and translational relevance of annexins becomes even more evident. Increasing evidence suggests that retinal alterations in AnxA1 and AnxA2 may serve as accessible biomarkers of CNS neurodegeneration, reinforcing the concept of the retina as a *window to the brain*. Moreover, annexin-derived pro-resolving peptides, such as Ac2-26, are emerging as promising therapeutic tools capable of modulating glial activity and restoring tissue homeostasis. Given the anatomical accessibility of the eye, the feasibility of localized ocular delivery further positions annexin-based interventions as a potential strategy for both diagnosis and therapy in PD and AD.

Finally, advancing our understanding of retinal glial dynamics particularly the contributions of microglia and pro-resolution astrocytes will be crucial to fully elucidate the neuroimmune mechanisms linking retinal and cerebral degeneration.

## Data Availability

No datasets were generated or analysed during the current study.
